# How to Read an EEG: Epileptiform Abnormalities

**DOI:** 10.1212/NE9.0000000000200265

**Published:** 2025-10-29

**Authors:** Kaley J. Marcinski Nascimento, Doyle Yuan, Rejean M. Guerriero, Lawrence J. Hirsch, Sándor Beniczky, Fábio A. Nascimento

**Affiliations:** 1 Department of Neurology, Washington University School of Medicine, St. Louis, MO;; 2 Department of Neurology, University of Texas Southwestern Medical Center, Dallas, TX;; 3 Peter O'Donnell Jr. Brain Institute, University of Texas Southwestern Medical Center, Dallas, TX;; 4 Department of Neurology, Yale School of Medicine, New Haven, CT; and; 5 Department of Clinical Neurophysiology, Aarhus University Hospital, Aarhus and Danish Epilepsy Centre, Dianalund, Denmark.

Accurately and reliably identifying epileptiform abnormalities is paramount in the care of patients with seizures, epilepsy, and nonepileptic paroxysmal events. We present an infographic (Figure) to help educators in teaching epileptiform abnormalities on non–critical care EEG.^1,2^ First, educators may introduce interictal epileptiform abnormalities, including epileptiform discharges operationally defined by the 6 IFCN criteria^e1^ and other epileptiform patterns such as paroxysmal fast activity, hypsarrhythmia, and temporal intermittent rhythmic delta activity.^1,e2^ Second, educators may review the definitions of potentially ictal abnormalities—namely brief potentially ictal rhythmic discharges.^2^ Finally, educators may discuss ictal patterns, ensuring that the concept of electrographic evolution is understood.^1,2^

**Figure:**
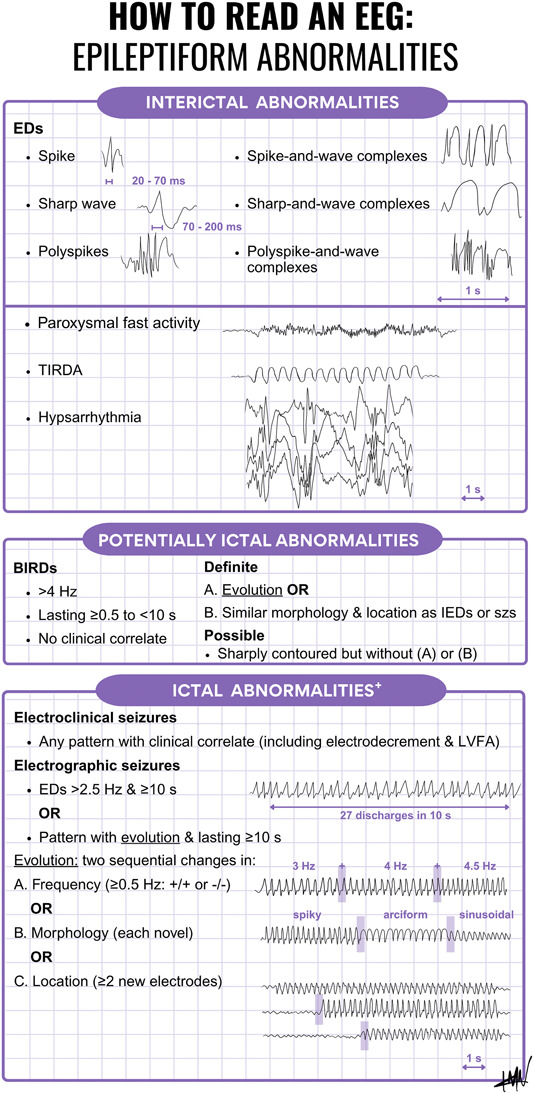
Epileptiform Abnormalities on EEG Epileptiform abnormalities include (A) interictal, (B) potentially ictal (BIRDs), and (C) ictal patterns. Evolution applies to both potentially ictal and ictal patterns.^2^ Note that the time scale varies and is shown as a purple double arrow. eTable 1 provides links to select examples. +, graphics in this section were adapted from Ref. 2. BIRDs = brief potentially ictal rhythmic discharges; ED = epileptiform discharge; IEDs = interictal epileptiform discharges; LVFA = low-voltage fast activity; szs = seizures; TIRDA = temporal intermittent rhythmic delta activity.
